# PB.21: Relationship between volumetric breast density and socioeconomic status

**DOI:** 10.1186/bcr3521

**Published:** 2013-11-08

**Authors:** L Samuels, SM Astley, A Maxwell, JC Sergeant, J Morris, M Wilson, P Stavrinos, DG Evans, A Howell, M Bydder

**Affiliations:** 1 University of Manchester, UK; 2 Nightingale Centre and Genesis Prevention Centre, University Hospital of South Manchester, UK; 3 University Hospital of South Manchester, UK

## Introduction

Women of higher socioeconomic status (SES) are at increased risk of developing breast cancer, but it is unclear to what extent this is mediated by differences in mammographic breast density, a well-established breast cancer risk factor. Here we explore the relationship between volumetric breast density and SES.

## Methods

Data from 6,398 perimenopausal/postmenopausal women undergoing routine NHSBSP screening were obtained from those consenting to the PROCAS (Predicting Risk Of Cancer At Screening) study. SES was assessed by obtaining the Index of Multiple Deprivation (IMD) associated with each participant's postcode. Volumetric breast density was measured using Volpara™. The association between volumetric breast density and IMD, adjusting for age, HRT use and BMI, was assessed using linear regression.

## Results

A very weak negative relationship was seen between volumetric breast density and IMD (B coefficient -0.023, *P *< 0.001), suggesting that increasing deprivation (lower SES) is associated with reduced breast density. A decreasing trend in IMD was seen with Volpara™ VDG breast density category (Table 1). This relationship was mainly driven by a negative association between SES and BMI, although unaffected by age or current HRT use.

**Table 1:** 

Volpara™ density grade (VDG)	Volpara™ breast density (%)	Mean IMD	Number (%) of women
1	<4.5	28.8	1923 (30.0%)
2	4.5 to 7.5	26.2	2765 (43.2%)
3	7.6 to 15.5	24.4	1505 (23.5%)
4	>15.5	21.4	205 (3.2%)

## Conclusion

SES (as measured by IMD) is not strongly associated with breast density. Established SES gradients in breast cancer risk are likely to be related to other SES-related factors, primarily BMI, rather than mammographic breast density.

